# Effects of noise on the internal resonance of a nonlinear oscillator

**DOI:** 10.1038/s41598-018-24383-2

**Published:** 2018-04-13

**Authors:** Damián H. Zanette

**Affiliations:** Centro Atómico Bariloche (CNEA) and Instituto Balseiro (UNCuyo), Consejo Nacional de Investigaciones Científicas y Técnicas, 8400 San Carlos de Bariloche, Río Negro, Argentina

## Abstract

We numerically analyze the response to noise of a system formed by two coupled mechanical oscillators, one of them having Duffing and van der Pol nonlinearities, and being excited by a self–sustaining force proportional to its own velocity. This system models the internal resonance of two oscillation modes in a vibrating solid beam clamped at both ends. In applications to nano– and micromechanical devices, clamped–clamped beams are subjected to relatively large thermal and electronic noise, so that characterizing the fluctuations induced by these effects is an issue of both scientific and technological interest. We pay particular attention to the action of stochastic forces on the stability of internal–resonance motion, showing that resonant oscillations become more robust than other forms of periodic motion as the quality factor of the resonant mode increases. The dependence on other model parameters —in particular, on the coupling strength between the two oscillators— is also assessed.

## Introduction

Vibrational dynamics of a solid beam is a classical problem in continuum mechanics —already dealt with by Euler and Bernoulli in the eighteenth century^1^— and has important applications in the construction of static and mobile structures, from machines to buildings of all kinds. Although the interest on this problem has hardly faded out and continues to attract the attention of researchers and designers^2^, its study has recently gained a renewed momentum in view of its relevance in the construction of micro– and nanomechanical systems^3^. In particular, time–keeping devices and sensors working at microscopic scales are expected to include oscillating elements consisting of tiny material beams, which are readily built during circuit printing and can be actuated by very small electric fields^4,5^.

A well–known phenomenon in the dynamics of vibrating beams is internal resonance, in which oscillations in a given mode (typically, the main oscillation mode) can excite other modes (typically, higher harmonics) if their frequencies are suitably syntonized with each other^6^. This is an effective mechanism for power transfer toward oscillation modes that might not be easy to excite by an external source, but which are able to efficiently couple with intermediary modes^7,8^. Recently, internal resonance has been proposed as a mechanism for frequency stabilization in nonlinear micromechanical oscillators formed by silica beams clamped at their two ends (clamped–clamped, or c–c, beams^9^). Vibrations of a c–c beam are well described by the Duffing equation, i.e. as a nonlinear oscillator with a cubic term in its restoring force^10,11^. Nonlinearity implies that the oscillation frequency and amplitude depend on each other (a–f effect^12^). This may hinder the application to time–keeping devices, as uncontrolled variations of amplitude —for instance, due to thermal or electronic noise— would bring about an undesired change in the frequency. However, it has been discovered that if the system operates within internal resonance, a regime develops in which the frequency becomes almost independent of the amplitude, and the a–f effect is thus suppressed^9^.

Micro– and nanomechanical devices usually work in highly fluctuating environments, where thermal and electronic noise is an unavoidable and significant dynamical ingredient. With respect to other forces involved in these systems, in fact, the relative effect of noise grows as the relevant length scales become smaller. Although the role of fluctuations in the operation of small–scale devices is widely acknowledged^12–14^, it does not seem that their action on the dynamics of micro– and nanomechanical oscillators has been systematically studied. In this paper, we numerically analyze the response of a vibrating c–c beam to noise, with emphasis in oscillations within the internal–resonance regime, although noise–induced transitions to other states are also considered. The effects of stochastic forces on a Duffing–like vibrating (macroscopic) beam has recently been characterized, both numerically and experimentally^15^, but the response of internal resonance to noise was not addressed in those previous studies. Here, we consider a c–c beam in a closed–loop configuration, whose oscillations are maintained by a self–sustaining force. This is the standard operational configuration in any time–keeping device^16^. The self–sustaining force is generated by conditioning a signal electronically read from the oscillation itself, and then reinjected as an “external” excitation. In our case, this force is proportional to the oscillation velocity delayed by a prescribed time. Such kind of feedback is able to generate self–sustained stationary oscillations if a van der Pol–like nonlinearity^17^ is also present in the damping force^18,19^. The reinjection of the self–sustaining force allows the mechanical oscillator to attain stable periodic motion, characteristic of a wide class of dynamical systems spanning from plasma physics^20^ to neurosciences^21^. The interaction between the resonant oscillation modes is described using a well–tested model^6,8,9^ in which modes are represented as mutually coupled one–dimensional oscillators.

The main goal of our work is to identify the most important factors that control the response of the oscillator to stochastic forces, determining the dominant dependences of the fluctuations induced by noise on the several parameters that define the system. Many of these parameters, in fact, are not necessarily amenable to experimental control, and numerical analysis is therefore necessary to assess their role in the present problem. In the next section, we introduce the two–oscillator model and discuss the main aspects of its dynamics in the absence of noise. Then, we describe our numerical methodology and present results under the action of stochastic forces. The last section is devoted to a qualitative discussion of the results and to a few concluding remarks.

## Two–oscillator model and noiseless dynamics

As a model for the interaction between two oscillation modes of a clamped–clamped (c–c) beam, we consider two linearly coupled one–dimensional oscillators, described by coordinates *x*_1_(*t*) and *x*_2_(*t*)^6^. The first oscillator is subjected to a self–sustaining force proportional to its own velocity at a delayed time, ${\dot{x}}_{1}(t-\tau )$, and is affected by a cubic (Duffing^11^) nonlinearity in the restoring force and a quadratic (van der Pol^17^) nonlinearity in the damping force^18,19^. This oscillator represent the main oscillation mode of the c–c beam. The second oscillator, which represents a higher–harmonic mode, is linear. Although moderate Duffing–like nonlinear response in higher–harmonic c–c beam vibration has been reported in experiments^9,22^, we show below that significant response of the higher–harmonic mode under conditions of internal resonance is limited to a narrow frequency interval, where higher–harmonic amplitude–frequency interdependence has no room to manifest itself. A linear higher–harmonic mode is therefore expected to satisfactorily describe the phenomenology focused on in the present contribution. Recently, the effects of higher–harmonic nonlinearity on the noiseless dynamics of vibrating c–c beams have been analyzed theoretically, showing that internal resonance preserves its main qualitative features over wide parameter ranges^23^.

The equations of motion derived for the model are

1 $${\ddot{x}}_{1}+{\gamma }_{1}\mathrm{(1}+4{x}_{1}^{2}){\dot{x}}_{1}+{\omega }_{1}^{2}{x}_{1}+\beta {x}_{1}^{3}=g{\dot{x}}_{1}(t-\tau )+j{x}_{2}+{\xi }_{1}(t),$$

2 $${\ddot{x}}_{2}+{\gamma }_{2}{\dot{x}}_{2}+{\omega }_{2}^{2}{x}_{2}=j{x}_{1}+{\xi }_{2}(t),$$

with *γ*_1,2_ the damping coefficients per unit mass, ${\omega }_{\mathrm{1,2}}^{2}$ the natural frequencies, *β* the Duffing coefficient, and *g* the gain of the self–sustaining force. The coordinates *x*_1,2_ have been rescaled in such a way that the coefficient of ${x}_{1}^{2}$ in the damping force of oscillator 1 has a prescribed value, and that the coupling strength *j* is the same for both oscillators. From now on, moreover, we fix *ω*_1_ ≡ 1 by a suitable choice of time units. The stochastic forces *ξ*_1,2_ are specified in the next section, at the stage of their numerical implementation.

In the noiseless case, *ξ*_1,2_ ≡ 0, approximate stationary solutions to the equations of motion can be found by the usual technique of proposing harmonic oscillations, ${x}_{1}(t)={A}_{1}\,\cos \,{\rm{\Omega }}t$ and ${x}_{2}(t)={A}_{2}\,\cos \,({\rm{\Omega }}t+\psi )$, and neglecting higher–harmonic contributions coming from nonlinear terms. For equations (1) and (2), this procedure yields

3 $$j{A}_{1}\,\sin \,\psi =-\,{\gamma }_{2}{\rm{\Omega }}{A}_{2},$$

4 $$j{A}_{1}\,\cos \,\psi =({\omega }_{2}^{2}-{{\rm{\Omega }}}^{2}){A}_{2},$$

5 $$j{A}_{2}\,\sin \,\psi ={\rm{\Omega }}[{\gamma }_{1}\mathrm{(1}+{A}_{1}^{2})-g\,\cos \,{\rm{\Omega }}\tau ]{A}_{1},$$

6 $$j{A}_{2}\,\cos \,\psi =\mathrm{(1}-{{\rm{\Omega }}}^{2}+\tilde{\beta }{A}_{1}^{2}-g\,{\rm{\Omega }}\,\sin \,{\rm{\Omega }}\tau ){A}_{1},$$

with $\tilde{\beta }=3\beta \mathrm{/4}$. These are equations for the amplitudes *A*_1,2_, the stationary frequency Ω, and the phase *ψ*.

**Figure 1:**
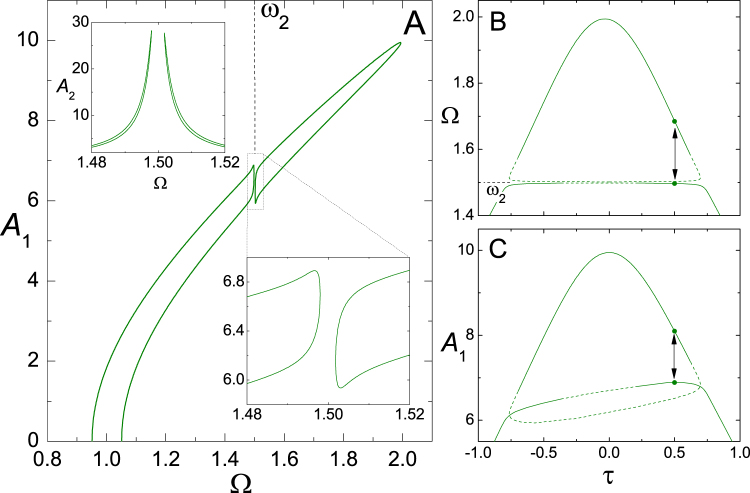
(**A**) Interdependence between the amplitude of oscillator 1 and the frequency of stationary oscillations, *A*_1_ vs Ω, for *γ*_1_ = 0.001, *γ*_2_ = 0.003, *ω*_2_ = 1.5, *β* = 0.04, *g* = 0.1, and ${j}^{2}=0.001$, as given by the solution to equations (3) to (6) when varying the time delay *τ* in the self–sustaining force. Lower–right inset: Close–up in the zone Ω ≈ *ω*_2_, where a gap in Ω occurs as a consequence of the resonance between the two oscillators. Upper–left inset: The amplitude of oscillator 2 as a function of the frequency, *A*_2_ vs Ω, around the gap. (**B**) The frequency of stationary oscillations as a function of the delay, Ω vs *τ*. Full (dashed) curves stand for stable (unstable) solutions. The vertical arrow points to the two stable solutions for *τ* = 0.5. (**C**) As in (**B**), for *A*_1_ vs *τ*.

The main panel of Fig. 1A shows the resulting interdependence of *A*_1_ vs Ω for *γ*_1_ = 0.001, *γ*_2_ = 0.003, *ω*_2_ = 1.5, *β* = 0.04, *g* = 0.1, and *j*^2^ = 0.001. The curve is parametrized by the time delay *τ*. We recognize the overall leaning shape of the Duffing resonance peak^11^. However, in contrast with the standard harmonically–forced Duffing oscillator, the present system does not have a low–amplitude solution for high frequencies^18^. This is a consequence of the linear dependence of the self–sustaining force on the velocity. Our main interest, nevertheless, lies in the zone where the frequency of stationary oscillations and the natural frequency of oscillator 2 are close to each other, Ω ≈ *ω*_2_. The lower–right inset in Fig. 1A is a close–up of that zone, where the resonance peak exhibits a gap in the frequency Ω^9,24^. Within the gap, no solution exists. The solutions at each side of the gap correspond to maximal oscillation amplitude for oscillator 2, as shown in the upper–left inset. These solutions represent the internal resonance of the two oscillation modes, for which the energy transfer from oscillator 1 to oscillator 2 is most efficient.

For sufficiently large *β*, *g*, and small *γ*_1_ —i.e. when the nonlinearity of oscillator 1 is well developed, with a markedly leaning, long, narrow Duffing peak— the width of the gap at Ω ≈ *ω*_2_ is mainly controlled by the damping of oscillator 2 and the strength of coupling between the oscillators, through the combination ${\rm{\Gamma }}={\gamma }_{2}\,{j}^{-2}$. For large values of this parameter, damping is too large and coupling is too weak to allow for resonance, and the gap is absent. As Γ becomes smaller, the gap is opened and initially widens but, when Γ decreases further, the width begins to decrease as well. For Γ small enough, the gap width is proportional to a power of this parameter. Taking the remaining parameters as in Fig. 1, the gap appears for ${\gamma }_{2}\approx 0.0077$ (Γ ≈ 7.7). However, for *γ*_2_ = 0.003 —as in the figure— its width is already in the zone where it varies proportionally to a power of Γ.

Figure 1B shows the solutions to equations (3) to (6) for the stationary frequency Ω as a function of the time delay *τ*, while Fig. 1C shows the corresponding solutions for *A*_1_ vs *τ*. Full and dashed curves indicate stable and unstable solutions, respectively. We stress that negative values of *τ* —which, naturally, cannot be realized in an experiment— are to be interpreted, for stationary harmonic motion, as positive time delays just below the oscillation period, i.e. generating phase shifts ${\rm{\Omega }}\tau \lesssim 2\pi$. The almost horizontal branches within the frequency gap at Ω ≈ *ω*_2_, in Fig. 1B, demonstrate the phenomenon of frequency stabilization associated with the internal resonance, discussed in the Introduction. We begin next section studying the transitions induced by noise between stable states inside and outside the gap, for fixed delay, illustrated by the vertical double arrows at *τ* = 0.5 both in Fig. 1B and C.

The stability of stationary solutions has been determined using a variation of the multiple–scale method^6^, which assumes that the time scale associated with the relaxation of oscillation amplitudes is much longer than the oscillation period. This is in fact the case, in particular, for experiments with micromechanical oscillators, whose weak damping (high quality factor, $Q\sim {10}^{4}-{10}^{5}$) insures a net separation between the two time scales. Around a stationary oscillation of frequency Ω, the amplitudes *A*_1,2_(*t*) and phases *ϕ*_1,2_(*t*) of the coordinates ${x}_{\mathrm{1,2}}(t)={A}_{\mathrm{1,2}}(t)\cos \,[{\rm{\Omega }}t+{\varphi }_{\mathrm{1,2}}(t)]$ evolve according to

7 $$2{\rm{\Omega }}{\dot{A}}_{1}={\rm{\Omega }}[g\,\cos \,{\rm{\Omega }}\tau -{\gamma }_{1}(1+{A}_{1}^{2})]{A}_{1}+j{A}_{2}\,\sin \,({\varphi }_{2}-{\varphi }_{1}),$$

8 $$2{\rm{\Omega }}{A}_{1}{\dot{\varphi }}_{1}=\mathrm{(1}-{{\rm{\Omega }}}^{2}+\tilde{\beta }{A}_{1}^{2}-g\,{\rm{\Omega }}\,\sin \,{\rm{\Omega }}\tau ){A}_{1}-j{A}_{2}\,\cos \,({\varphi }_{2}-{\varphi }_{1}),$$

9 $$2{\rm{\Omega }}{\dot{A}}_{2}=-\,{\gamma }_{2}{\rm{\Omega }}{A}_{2}-j{A}_{1}\,\sin \,({\varphi }_{2}-{\varphi }_{1}),$$

10 $$2{\rm{\Omega }}{A}_{2}{\dot{\varphi }}_{2}=({\omega }_{2}^{2}-{{\rm{\Omega }}}^{2}){A}_{2}-j{A}_{1}\,\cos \,({\varphi }_{2}-{\varphi }_{1}\mathrm{)}.$$

As can be verified by direct inspection, the stationary solutions to these equations coincide with the solutions to equations (3) to (6) with *ψ* = *ϕ*_2_ − *ϕ*_1_. Within the multiple–scale approximation, linearization of equations (7) to (10) makes it possible to decide on the stability of the stationary oscillations. Also, this analysis provides the relaxation rates toward the stationary state which, as shown in the next section, play a role in the response of the system to the effects of noise.

## Response to stochastic forces

Inside the internal–resonance gap, the close proximity of a stable and an unstable solution for fixed *τ*, as illustrated by Fig. 1B (for instance, for *τ* = 0.5), naturally rises the question on how robust against the effects of noise the system is, if prepared to oscillate in the stable solution. Is the system able to resist stochastic fluctuations and remain inside the gap, or will it immediately migrate to the stable oscillation of higher frequency which, for the same value of *τ*, lies outside the gap? This question becomes particularly relevant if, as advanced in the Introduction, internal resonance is sought to be exploited as a way of stabilizing the frequency of the nonlinear oscillator against fluctuations of the oscillation amplitude.

To address this problem, we have solved equations (1) and (2) numerically, using a standard explicit second–order Runge–Kutta scheme adapted to stochastic differential equations^25^. The stochastic forces *ξ*_1,2_(*t*) have been implemented by interpreting the equations for the velocities ${v}_{\mathrm{1,2}}(t)={\dot{x}}_{\mathrm{1,2}}(t)$ in the Ito form, *dv*_*i*_ = *F*_*i*_*dt* + *σ*_*i*_*dtW*_*i*_, where *F*_*i*_ are the deterministic forces and *dW*_*i*_ are differential Wiener processes with 〈*dW*_*i*_〉 = 0 and $\langle d{W}_{i}^{2}\rangle =dt$. The coefficients *σ*_*i*_ weight the strength of noise. The stochastic differential equation is thus discretized (to the first order) as ${\rm{\Delta }}{v}_{i}={F}_{i}{\rm{\Delta }}t+{\sigma }_{i}{({\rm{\Delta }}t)}^{\mathrm{1/2}}{\zeta }_{i}$^26^. In our calculations, successive values of the stochastic variable *ζ*_*i*_ have been drawn from a Gaussian distribution with zero mean and unitary variance, using the pseudorandom number generator RANDGEN^27^.

**Figure 2:**
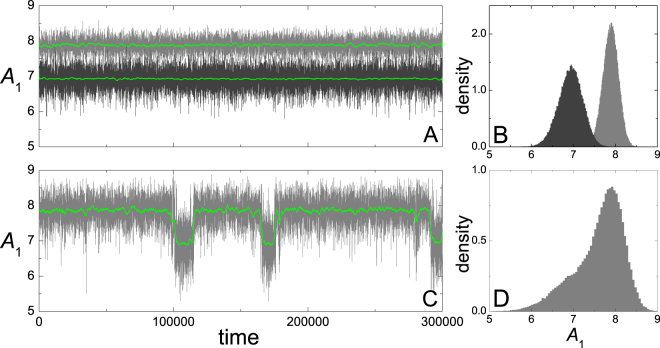
Time signals for the amplitude of oscillator 1 under the action of stochastic forces, recorded as explained in the text, for *γ*_1_ = 0.001, *γ*_2_ = 0.004, *ω*_2_ = 1.5, *β* = 0.04, *g* = 0.1, ${j}^{2}=0.001$, and *τ* = 0.5. (**A**) Two runs with *σ*_1_ = 0.2, *σ*_2_ = 0, and initial conditions in the vicinity of each stable solution of the noiseless system. (**B**) Normalized histograms for the values of *A*_1_ from each temporal series in (**A**). (**C**,**D**) As in (**A**,**B**), for a single run with *σ*_1_ = 0.3. Green curves are running averages over 300 successive values of *A*_1_.

For the numerical runs, we have chosen initial conditions that led, in the absence of noise, to a selected stable solution –inside or outside the gap. We have first run the system without noise until it closely approached the selected solution and, then, the stochastic forces were turned on. Along each realization, we have recorded the successive local maxima of the coordinates *x*_1,2_(*t*), as a measure of the amplitudes *A*_1,2_ affected by noise. In the presentation of our results, however, we focus on the behavior of *A*_1_, since we expect the amplitude of the main oscillation mode of the c–c beam —represented in our model by oscillator 1— to be most readily accessible to experimental measurements^7–9^. Figure 2 shows a few typical realizations for the amplitude of oscillator 1, with two values of *σ*_1_ and *σ*_2_ = 0. The other parameters are *γ*_1_ = 0.001, *γ*_2_ = 0.004, *ω*_2_ = 1.5, *β* = 0.04, *g* = 0.1, and *j*^2^ = 0.001, with time delay *τ* = 0.5. In Fig. 2A and B, *σ*_1_ = 0.2. Figure 2A depicts two time series for *A*_1_, starting from initial conditions in the vicinity of either stable solution, inside (dark gray, lower series) and outside (light gray, upper series) the gap. Green lines show time averages over 300 successive values of *A*_1_. In Fig. 2B, normalized histograms of the values of *A*_1_ in each series are shown. We see that for, this noise level, both solutions fluctuate around well–defined values, with a larger dispersion for the oscillation inside the gap. In Fig. 2C and D, the stochastic force on oscillator 1 has been increased to *σ*_1_ = 0.3. Here, from any initial condition in the vicinity of the stable solutions, the amplitude *A*_1_ exhibits occasional transitions between the two states. For these parameters, however, the signal is most of the time closer to the upper state. This is also evident from the corresponding histogram.

**Figure 3:**
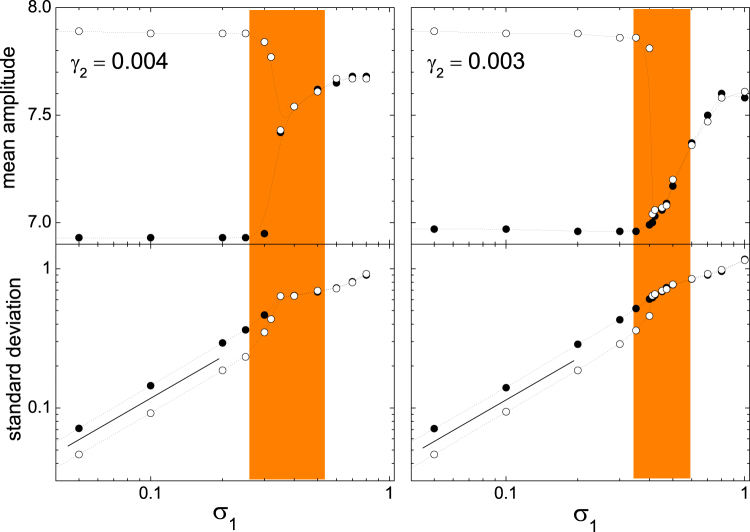
Mean value (log–linear scales) and standard deviation (log–log scales) of the amplitude *A*_1_, measured from numerical runs like those of Fig. 2, as functions of the noise strength *σ*_1_, for *γ*_2_ = 0.004 (left) and *γ*_2_ = 0.003 (right). All the other parameters are the same as in Fig. 2. Full and empty dots stand for initial conditions that, in the noiseless case, lead to stable states inside and outside the gap, respectively. Measurements correspond to averages over 2 × 10^5^ to 5 × 10^6^ time units. Dotted curves are spline interpolations, plotted as a guide to the eye. Straight segments between the two standard deviation datasets for small *σ*_1_ have slope one. Vertical stripes correspond to the intervals of *σ*_1_ where *A*_1_ performs occasional transitions between the states inside and outside the gap, as in Fig. 2C.

A quantitative characterization of the fluctuations in the oscillation amplitude is given by the mean value and standard deviation of *A*_1_ along the realizations described in the preceding paragraph. The leftmost panels in Fig. 3 show these averages for the same parameters of Fig. 2, as functions of the noise intensity *σ*_1_. Full and empty dots correspond to initial conditions that lead to the stable states inside and outside the internal resonance gap, respectively. For weak noise, $\sigma \lesssim 0.25$, the corresponding mean values of *A*_1_ are well separated from each other, while the standard deviations grow linearly with *σ*_1_, as demonstrated by the straight line between the two datasets. On the other hand, for sufficiently strong noise, $\sigma \mathop{ > }\limits_{ \tilde {}}0.5$, the two datasets collapse for both the mean amplitude and the standard deviation. Under the effect of such large fluctuations, the amplitude randomly visits a large interval around *A*_1_ ≈ 7.7, comprising both stable states, with a standard deviation that still grows proportional to *σ*_1_. For intermediate noises, in the zone highlighted in Fig. 3 by the vertical stripe, the amplitude shows transitions between the two states, as illustrated in Fig. 2C, with a prevalence of the higher–amplitude solution. In the leftmost half of the stripe, it is still possible to measure clearly defined values for the mean amplitude and the standard deviation for each state, as the system spends well–separated periods in their respective vicinities (as illustrated in Fig. 2C). As *σ*_1_ grows, however, the separation becomes less defined, and the two quantities merge into single values. Within this zone, the standard deviation clearly abandons its linear dependence on *σ*_1_.

The panels to the right of Fig. 3 show the same results as to the left but for a lower value of the damping coefficient of oscillator 2, *γ*_2_ = 0.003. While the overall behavior is the same as for *γ*_2_ = 0.004, now it is the state of lower amplitude which prevails over the other one during the transitions within the vertical stripe. Thus, the system spends more time inside the internal resonance gap. Note also that the vertical stripe is narrower than for larger *γ*_2_, and that the value of *σ*_1_ at which the two datasets merge is slightly shifted to the right, from *σ*_1_ ≈ 0.35 to 0.4. As *γ*_2_ decreases further, the prevalence of the stationary state inside the gap grows. For *γ*_2_ = 0.002 it is already impossible to find values of *σ*_1_ where the amplitude shows transitions between the two states, and the threshold for which the signal merge drops to *σ*_1_ ≈ 0.2. For *γ*_2_ = 0.001 (=*γ*_1_), although the oscillation outside the gap still is a stable solution to equations (7) to (10), very weak noise (${\sigma }_{1}\lesssim {10}^{-3}$) is able to induce the transition to the state inside the gap, which is later never abandoned. For small values of *γ*_2_, therefore, the resonant oscillation is virtually the only accessible stationary state under the action of noise.

**Figure 4:**
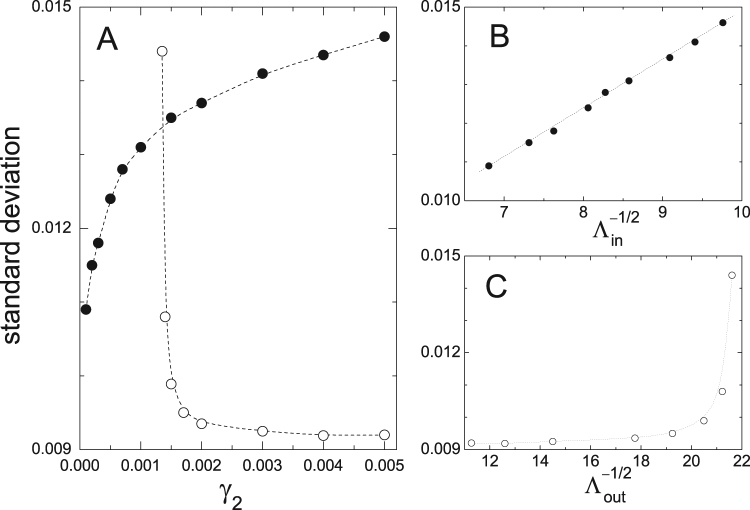
(**A**) Standard deviation of the amplitude *A*_1_ as a function of the damping coefficient *γ*_2_ of oscillator 2, for a noise strength *σ*_1_ = 0.01. Full and empty dots correspond, respectively, to fluctuations around the states inside and outside the resonance gap. Dotted curves are spline interpolations, plotted as a guide to the eye. (**B**,**C**) The same results as functions of the square root of the typical relaxation times around each state, estimated as minus the inverse real parts of the relevant eigenvalues of equations (7) to (10).

In order to gain some insight on the origin of this behavior, we have measured the standard deviation of *A*_1_ around the stationary states as a function of *γ*_2_. Figure 4A shows the results for a fixed noise intensity *σ*_1_ = 0.01. Full dots corresponds to fluctuations around the state inside the gap. Deviations in the amplitude decrease as *γ*_2_ becomes smaller, indicating that the resonant oscillation is more robust to noise for smaller damping of oscillator 2. The state outside the gap (empty dots), in contrast, has the opposite trend. In particular, the standard deviation exhibits a sharp growth for ${\gamma }_{2}\mathop{ > }\limits_{ \tilde {}}0.001$, which is compatible with the fact that the oscillators migrate to the resonant state even for very small noises.

In a naive picture, we expect the standard deviation of *A*_1_ to be proportional to the noise strength, and to the square root of a typical time of relaxation toward equilibrium. These are in fact the two main ingredients that determine the size of fluctuations induced by noise on a system at equilibrium in a harmonic potential well (Ornstein–Uhlenbeck process^28^). The lower panels of Fig. 3 make it clear that, for weak noise, the standard deviation is indeed proportional to *σ*_1_. The damping coefficient *γ*_2_, on the other hand, controls —along with all the other parameters— the eigenvalues of equations (7) to (10) at their stable fixed points, which can in turn be used to estimate the relaxation times toward equilibrium^17^. The eigenvalues relevant to this estimation have the form ${\mu }_{{\rm{in}},{\rm{out}}}=-\,{{\rm{\Lambda }}}_{{\rm{in}},{\rm{out}}}\pm i{\nu }_{{\rm{in}},{\rm{out}}}$ where the subindices “in,out” refer to the states inside and outside the resonance gap. The respective relaxation times are given by minus the inverse of their real parts, ${{\rm{\Lambda }}}_{{\rm{in}},{\rm{out}}}^{-1}$. Figure 4B and C show the standard deviation of *A*_1_ around each stable state as a function of the corresponding values of ${{\rm{\Lambda }}}_{{\rm{in}},{\rm{out}}}^{-\mathrm{1/2}}$. While for the resonant state we find a linear relation, as expected on the basis of the above argument, for the state outside the gap the dependence is far from linear. This indicates that the naive picture of a response to noise controlled just by linear relaxation is not valid for the stationary state outside the resonance gap. Plausibly, nonlinear effects are involved in the process by which this state looses stability under the action of the stochastic force.

### Dependence on other parameters

In the preceding paragraphs, we have analyzed the response to noise of the two–oscillator model as a function of the damping coefficient of oscillator 2 —which is one of the parameters that controls the width of the resonance gap— and of the strength of the stochastic force acting on oscillator 1. In this subsection, we briefly report on the effects of varying the remaining parameters.

**Figure 5:**
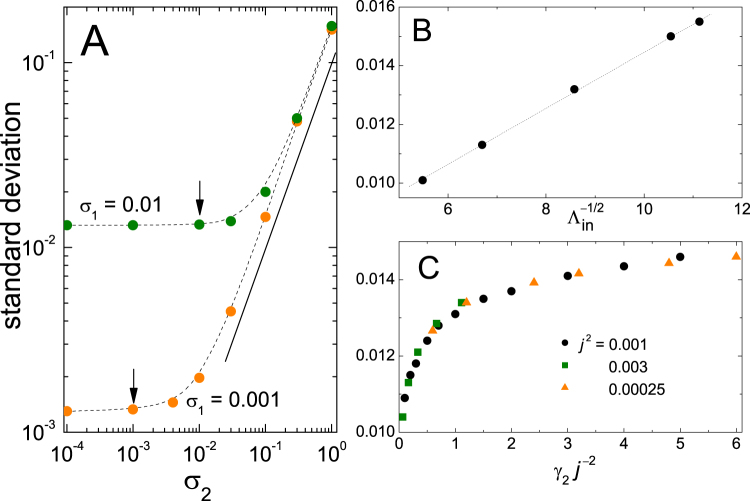
(**A**) Standard deviation of the amplitude *A*_1_ around the stable resonant oscillation as a function of the strength of the stochastic force acting on oscillator 2, for two values of *σ*_1_. For each set, the vertical arrows indicate the horizontal position of *σ*_1_. The straight full line has one, and the dashed curves are splines plotted as a guide to the eye. Other parameters are for *γ*_1_ = *γ*_2_ = 0.001, *ω*_2_ = 1.5, *β* = 0.04, *g* = 0.1, *j*^2^ = 0.001, and *τ* = 0.5. (**B**) Standard deviation of *A*_1_ as a function of the typical relaxation time ${{\rm{\Lambda }}}_{{\rm{in}}}^{-\mathrm{1/2}}$ (see text and Fig. 4B) for varying *γ*_1_. The other parameters are as in (**A**), with *σ*_1_ = 0.01 and *σ*_2_ = 0. (**C**) Standard deviation of *A*_1_ for three values of *j*^2^, as a function of the combination ${\rm{\Gamma }}={\gamma }_{2}\,{j}^{-2}$. Other parameters are as in (**B**), with ${\gamma }_{1}=0.001$.

In the first place, we have numerically solved equations (1) and (2) for two fixed values of *σ*_1_, varying the strength of the stochastic force on oscillator 2, *σ*_2_, with all the other parameters as in Figs 2–4 and *γ*_2_ = 0.001. Naturally, in an experimental realization of the c–c oscillator, the noise amplitudes *σ*_1_ and *σ*_2_ are not independent parameters, but are related by the continuum dynamics of the beam. In our numerical simulations, however, we have the freedom of varying each of them separately. Figure 5A shows the standard deviation of the amplitude *A*_1_ around the stable state inside the resonance gap for *σ*_1_ = 0.01 and *σ*_1_ = 0.001 as a function of *σ*_2_. In both cases, we find two well–defined regimes. For ${\sigma }_{2}\lesssim {\sigma }_{1}$ and below, the standard deviation does not depend on *σ*_2_, and its value coincides, up to small variations, with that obtained for *σ*_2_ = 0. This indicates that, in this regime, the effect of noise is dominated by *σ*_1_. However, as the stochastic force on oscillator 2 grows stronger, ${\sigma }_{2}\mathop{ > }\limits_{ \tilde {}}10{\sigma }_{1}$ and above, the fluctuations in the amplitude become proportional to *σ*_2_, indicating a prevalence with respect to the noise on oscillator 1. Note that, due to the linearity of the equation of motion for oscillator 2, its amplitude should fluctuate proportionally to *σ*_2_. The coordinate *x*_2_, in turn, enters the equation for oscillator 1 weighted by the coupling strength *j*. Therefore, we expect that the stochastic force on oscillator 2 contributes fluctuations proportional to the product $j{\sigma }_{2}$ to the amplitude of oscillator 1.

We have already mentioned that the nonlinear coefficient *β* and the self–sustaining force gain *g* determine the form of the Duffing resonance peak but, as long as they are large enough, they do not affect the zone of the resonance gap. Therefore, they are not expected to influence the response to noise of the stable resonant oscillations. The same holds for the frequency *ω*_2_, which defines the position of the resonance gap, as long as it lies far enough from the end of the resonance peak. Moreover, the occurrence of internal resonance does not require that *ω*_2_ has any special relation to *ω*_1_. On the other hand, the damping coefficient *γ*_1_ controls the width of the peak along its whole length. To assess the effect of varying this coefficient, we have solved equations (1) and (2) with the same parameters as in Fig. 5A, fixing ${\sigma }_{1}=0.01$ and *σ*_2_ = 0, for various values of *γ*_1_. In Fig. 5B we show the standard deviation of oscillator 1 in the stable state inside the gap as a function of ${{\rm{\Lambda }}}_{{\rm{in}}}^{-\mathrm{1/2}}$ (see Fig. 4B), with ${{\rm{\Lambda }}}_{{\rm{in}}}$ calculated as a function of *γ*_1_ from the eigenvalues of equations (7) to (10). We verify that the linear dependence reported in Fig. 4C for varying *γ*_2_ subsists upon variation of *γ*_1_.

Finally, we focus on the coupling strength *j*. In our discussion of the size of the resonance gap, we pointed out that its width is defined by the combination ${\rm{\Gamma }}={\gamma }_{2}\,{j}^{-2}$. Conjecturing that this combination might also control the response to noise inside the gap, we have solved equations (1) and (2) with the same parameters as before, fixing *γ*_2_ and varying *j*^2^. Figure 5C shows the standard deviation of oscillator 1 inside the gap as a function of Γ. The collapse of the results onto a single curve confirms that our conjecture is valid for these parameter sets.

## Discussion

We have studied the response to stochastic forces of a system of two coupled mechanical oscillators, which model the internal resonance of a clamped–clamped solid beam. The coexistence of two stable oscillatory states —one of them representing the resonant oscillation— raises the question of their relative stability under the action of noise. Answering this question is relevant to possible applications of internal resonance, specifically, as a way of stabilizing the oscillation frequency in micromechanical nonlinear oscillators^9^.

**Figure 6:**
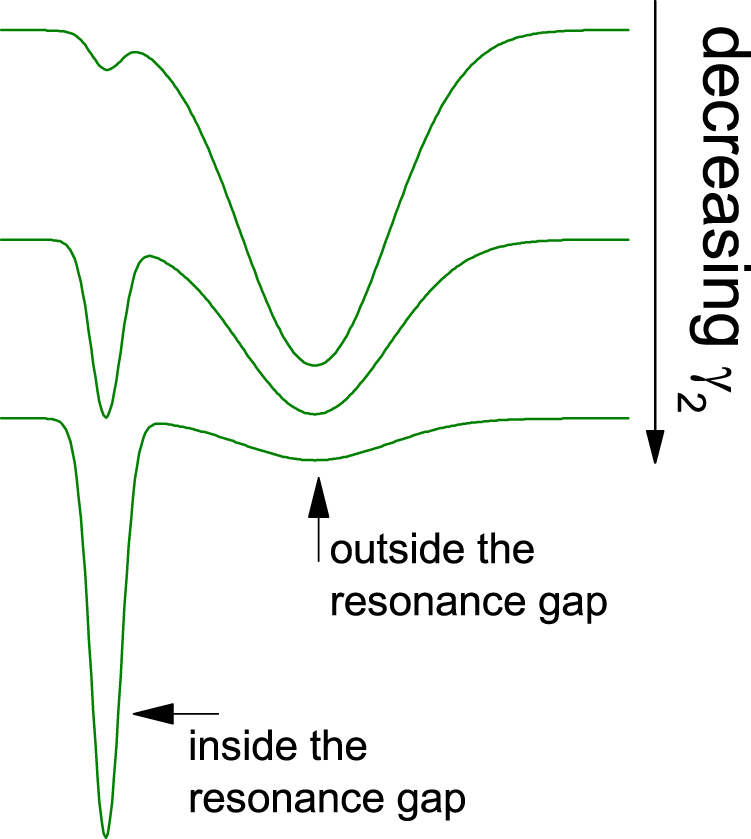
Qualitative picture, in terms of a fictitious nonequilibrium potential represented by the curves, of the relative stability of the stable oscillations inside and outside the resonance gap, when varying the damping coefficient *γ*_2_ of oscillator 2. The two minima and the intermediate maximum stand, respectively, for stable and unstable oscillations.

We have shown that the relative stability of the two coexisting oscillations depends on the parameters that, in turn, control the width of the frequency gap induced by internal resonance —in particular, the damping coefficient of the resonant mode. Due to its dimensionality, it is not possible to formally describe our model as a system randomly evolving under the action of noise on a potential landscape^28^. However, from our numerical results, a qualitative picture emerges in which, as the damping coefficient of the resonant mode decreases (i.e. its quality factor grows), the resonant oscillation sharply strengthens its stability with respect to the other oscillation, in spite of the increasingly closer vicinity of an unstable state across the gap. This is schematically shown in Fig. 6, where stable and unstable oscillations are respectively pictured as the minima and maxima of a (fictitious) nonequilibrium potential. It is worth mentioning that, under experimentally controlled conditions, the damping coefficients of each mode can, to a certain extent, be varied independently from each other^29^. Here we have moreover considered variations of the coupling strength between the modes, which also influences the gap width.

As a final comment, let us point out that our selection of a self–sustaining force proportional to the oscillation velocity is just but one of the possible feedbacks by which a micromechanical oscillator can be excited into stationary motion. In our case, the choice was motivated by convenience in the numerical implementation. Other experimentally tested feedbacks include self–sustaining forces with controlled maximal strength^9^, and proportional to the oscillation amplitude^19^. Although the zones of parameter space where stationary motion is stable or unstable may vary depending on the kind of excitation, internal resonance is expected to occur in all of them, so that our present conclusions should apply —at least, qualitatively— to those other realizations of the self–sustaining feedback.
